# Impact of Community-Based Clinical Breast Examinations in Botswana

**DOI:** 10.1200/GO.20.00231

**Published:** 2021-01-06

**Authors:** Michael Dykstra, Brighid Malone, Onica Lekuntwane, Jason Efstathiou, Virginia Letsatsi, Shekinah Elmore, Cesar Castro, Neo Tapela, Scott Dryden-Peterson

**Affiliations:** ^1^ Beth Israel Deaconess Medical Center, Boston, MA; ^2^ Botswana-Harvard AIDS Institute Partnership, Gaborone, Botswana; ^3^ Bokamoso Private Hospital, Gaborone, Botswana; ^4^ Journey of Hope Botswana, Gaborone, Botswana; ^5^ Massachusetts General Hospital, Boston, MA; ^6^ Botswana Ministry of Health and Wellness, Gaborone, Botswana; ^7^ Department of Radiation Oncology, University of North Carolina, Chapel Hill, NC; ^8^ University of Oxford, Oxford, UK; ^9^ Brigham and Women's Hospital, Boston, MA; ^10^ Harvard School of Public Health, Boston, MA

## Abstract

**PURPOSE:**

We evaluated a clinical breast examination (CBE) screening program to determine the prevalence of breast abnormalities, number examined per cancer diagnosis, and clinical resources required for these diagnoses in a middle-income African setting.

**METHODS:**

We performed a retrospective review of a CBE screening program (2015-2018) by Journey of Hope Botswana, a Botswana-based nongovernmental organization (NGO). Symptomatic and asymptomatic women were invited to attend. Screening events were held in communities throughout rural and periurban Botswana, with CBEs performed by volunteer nurses. Individuals who screened positive were referred to a private tertiary facility and were followed by the NGO. Data were obtained from NGO records.

**RESULTS:**

Of 6,120 screened women (50 men excluded), 452 (7.4%) presented with a symptom and 357 (5.83%) were referred for further evaluation; 257 ultrasounds, 100 fine-needle aspirations (FNAs), 58 mammograms, and 31 biopsies were performed. In total, 6,031 were exonerated from cancer, 78 were lost to follow-up (67 for ≤ 50 years and 11 for > 50 years), and 11 were diagnosed with cancer (five for 41-50 years and six for > 50 years, 10 presented with symptoms). Overall breast cancer prevalence was calculated to be 18/10,000 (95% CI, 8 to 29/10,000). The number of women examined per breast cancer diagnosis was 237 (95% CI, 126 to 1910) for women of age 41-50 years and 196 (95% CI, 109 to 977) for women of age > 50 years. Median time to diagnosis for all women was 17.5 [1 to 32.5] days. CBE-detected tumors were not different than tumors presenting through standard care.

**CONCLUSION:**

In a previously unscreened population, yield from community-based CBE screening was high, particularly among symptomatic women, and required modest diagnostic resources. This strategy has potential to reduce breast cancer mortality.

## INTRODUCTION

Breast cancer incidence and mortality is rising rapidly in sub-Saharan Africa.^1,2^ Because of decreased mortality from infections and birth complications and changing demographic factors including decreased and delayed childbearing, less physical activity, higher levels of obesity, and increased alcohol consumption, deaths from breast cancer have nearly doubled in the past two decades in southern Africa.^3^ Case fatality rates are high in Africa largely because of advanced stage at presentation and inadequate access to diagnostics and treatment (surgery, radiotherapy, and chemotherapy).^2-5^ In response, countries and nongovernmental organizations (NGOs) are seeking effective strategies for early detection of breast cancer.^6,7^

**CONTEXT**

**Key Objective**
We sought to estimate the yield and the diagnostic resources required for clinical breast examination (CBE) campaigns in previously unscreened African populations.
**Knowledge Generated**
Among 6,120 women examined through community-based, nongovernmental organization CBE campaigns in rural Botswana, 6.4% of women of age 40 years and younger and 5.2% of women of age 40 years and older required additional testing for detected abnormalities. Eleven cancers were diagnosed, all among women older than 40 years, with 217 women older than 40 years examined per cancer diagnosis.
**Relevance**
Community-based CBE required diagnostic resources feasible in many African countries and efficiently identified untreated cancers, particularly among women older than 40 years.

First, breast cancer awareness and well-defined referral pathways for diagnostic evaluation are essential for early diagnosis.^8-10^ Breast cancer screening with clinical breast examinations (CBEs) has been shown to reduce the stage at which cancer is diagnosed in cluster randomized control trials in India.^11,12^ Other studies have also demonstrated the potential for CBE and algorithm training to increase detection and decrease stage of breast cancers, which is expected to improve mortality.^7,13-15^ Given these preliminary results and the infeasibility of large-scale mammography screening in low- and middle-income countries (LMICs), a number of governments and nonprofit organizations have introduced CBE breast cancer screening programs in LMICs globally.^7^ The WHO does not currently endorse any strategy of population breast cancer screening in LMICs, finding mammography not cost effective in that context,^16^ and other technologies such as piezoelectric-based handheld devices require further study in LMIC contexts prior to widespread clinical implementation.^17,18^ However, the WHO suggests CBE screening could be useful in settings it is shown to be effective and sustainable and where appropriate diagnostic and treatment services are available.

Botswana, a middle-income country with a predominantly rural population, endorsed routine breast screening by CBE in the 2016 core primary care guidelines for women of age 40-69 years.^19^ With multimodality breast cancer treatment available free of charge for citizens, it is possible that if a CBE program reduces stage at presentation, it could lead to a reduction in mortality. The unknown prevalence of breast abnormalities and breast cancers requiring diagnosis and treatment also impedes planning efforts for screening programs.^16^ Using records from a large CBE initiative by a Botswana-based NGO, we sought to determine screening uptake, prevalence of breast abnormalities, number screened per breast cancer diagnosis, and clinical resources required to achieve diagnoses. Secondary analyses included proportion of women completing diagnostic evaluation and time to diagnosis. Findings may inform planning of national CBE screening programming in Botswana and similar settings.

## METHODS

We performed a retrospective review of records from a CBE-based breast cancer screening program that was conducted by a Botswana-based NGO, Journey of Hope Botswana (JOHB). Established in 2010, this organization promotes breast cancer awareness in the country through events and media campaigns.

### Screening Event Structure

**FIG 1:**
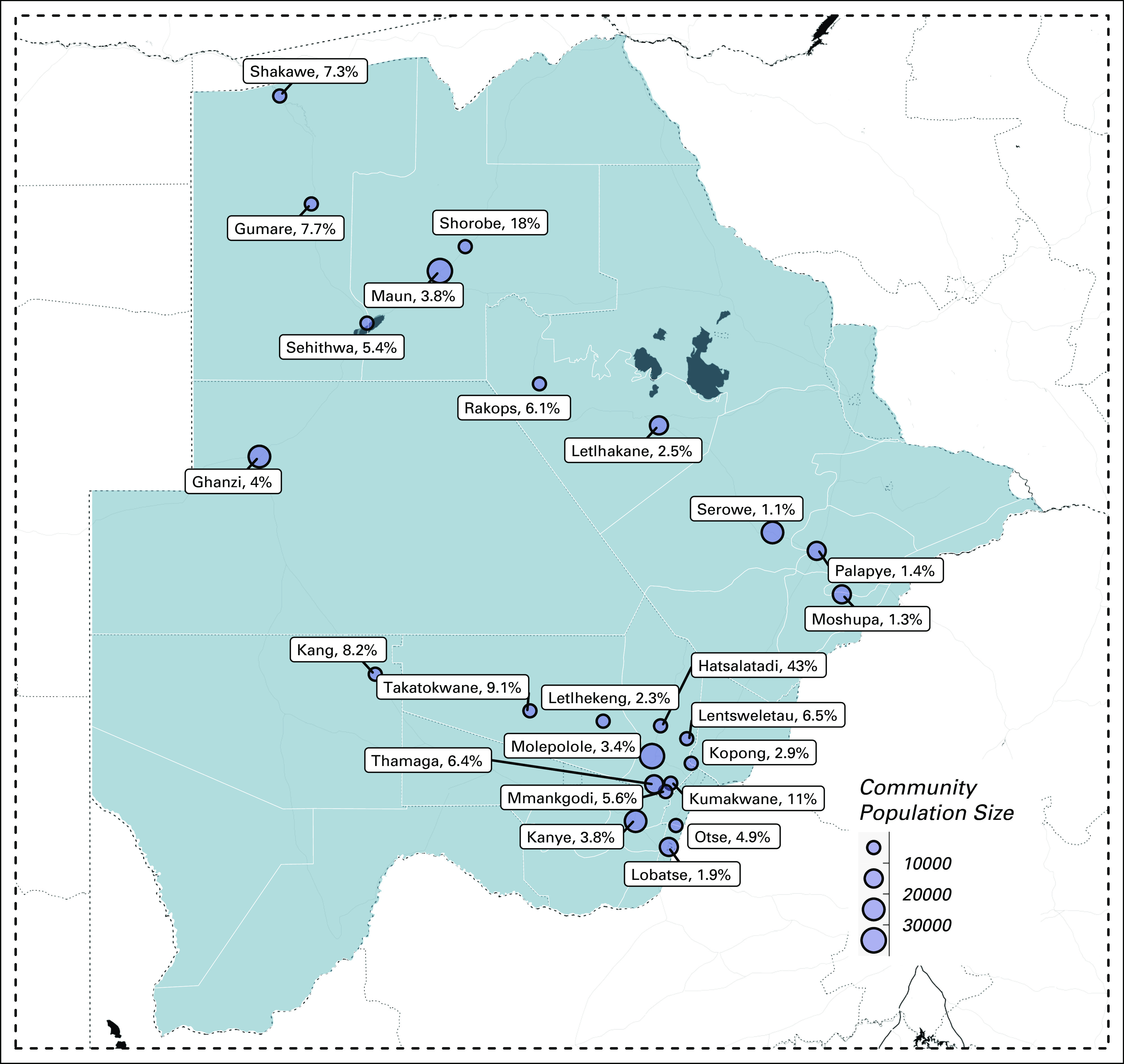
Town size is represented by dot size (see scale for details) on this map, and each town is labeled by name and the percentage of women older than the age of 20 years who were screened in each community.

An annual community-based Big Ride screening event has been conducted by JOHB since 2010, with 1,000 to 2,000 women screened each year. Supported by philanthropy from local businesses, JOHB volunteers convoy in pink vehicles to day-long screening events in five to seven rural and periurban communities. Locations for screening events are chosen based on perceived need, geographic proximity to one another, and support from local chiefs and clinic staff. See Figure 1 for a map demonstrating the towns visited during the years included in this analysis. Pre-event sensitization is supported by community leaders, local clinic staff, and via social media. Attendance is also encouraged through creation of a festive environment and distribution of gifts including bras and t-shirts.

Annually, prior to screening events, nurses are trained to perform CBEs by a general practice physician using models and healthy patient volunteers. Single-day screening events were held inside local public clinics or outdoor tents, where symptomatic or asymptomatic women can present for evaluation. A volunteer nurse evaluates patients first. Patients with a detected breast abnormality on this examination are referred to an on-site general practitioner for determination of need for diagnostic referral. Services at the events have expanded over time with introduction of on-site fine-needle aspiration in 2015 and breast ultrasound examination in 2018. The diagnostic algorithm was developed by a general practitioner from the NGO. Patients with suspicious breast abnormalities were referred to a private tertiary hospital located in the capital, where they were evaluated by ultrasound with or without mammogram. Patients were further referred for a core breast biopsy if tests were suspicious for malignancy. Several patients received core biopsies by a physician at the event prior to other evaluation. JOHB covered costs for transportation, accommodation, and testing. When breast cancer diagnoses were made, patients were referred to a public tertiary referral hospital for multimodality treatment. JOHB continued following up with patients throughout treatment but did not cover costs of transportation or accommodations after diagnosis.

### Data Collection

Records from 28 screening events from 2015 to 2018 were abstracted from JOHB records. Patients’ age was recorded in 10-year increments (≤ 20, 21-30, 31-40, 41-50, 51-60, and > 60 years). For referral outcomes, all information was confirmed by the physician who oversaw the events and follow-up. Dates of diagnostic test results were obtained directly from JOHB paper records. Cancer was defined as any biopsy-proven breast cancer or ductal carcinoma in situ. Clinical tumor stage (T-stage) was determined using NGO imaging records of tumor dimensions. Nodal and distant metastases (N and M stages) were determined based on symptom review, clinical examination, chest x-ray, and abdominal ultrasound. No patients were contacted directly for this study. The institutional review boards of the Botswana Ministry of Health and Wellness and the Harvard T.H. Chan School of Public Health approved the study and waived requirement for informed consent.

Follow-up costs were estimated using rates charged to patients or insurance by the private hospital for standard procedures, although costs were waived for the NGO. Costs of the screening events or patient navigation were not included. Community population for each town was estimated from the 2011 census in Botswana, assuming 3% annual growth and consistent age-sex distributions.

### Stage Comparison Cohort

The T-stage at diagnosis through JOHB was compared with those enrolled in a prospective cohort study including all patients with biopsy-proven cancers who present to any oncology referral center in Botswana. This cohort has been described in prior studies regarding other types of cancer in Botswana.^20,21^ Patients from this cohort were matched in a 4:1 ratio to JOHB patients based on age and district.

### Statistical Analysis

The primary analytic objectives were to determine screening uptake, proportions of screened women with a positive CBE, and number screened per breast cancer diagnosis. End points were summarized in three age categories: 20-40, 41-50, and women > 50 years. The binomial distribution was used to calculate 95% CI of assessed proportions. The Kaplan-Meier method was used to estimate time to final diagnosis, and Greenwood’s formula used to calculate 95% CIs.^22^ Comparisons of subgroups used Wilcoxon rank-sum and Fisher’s exact tests for continuous and categorical measures, respectively. All tests were two-tailed with a significance level of 0.05. Analysis was performed using R.^23^

## RESULTS

### Screening Participation

A total of 6,120 women and 50 men were screened with CBE from 2015 to 2018 (Table 1). Because all women were encouraged to come, 452 (7.4%) of attendees came with a breast-related complaint, including pain, lumps, discharge, or rash, but most were asymptomatic. The majority of women were younger than 40 years. The events were able to reach 3.68% of women of age 20-40 years, 4.15 % of 40-50 years, and 2.98% of women older than 50 years residing in the catchment area. Reach of CBE screening was variable between communities and was dependent on community size (Fig 1, Data Supplement).

**TABLE 1:**
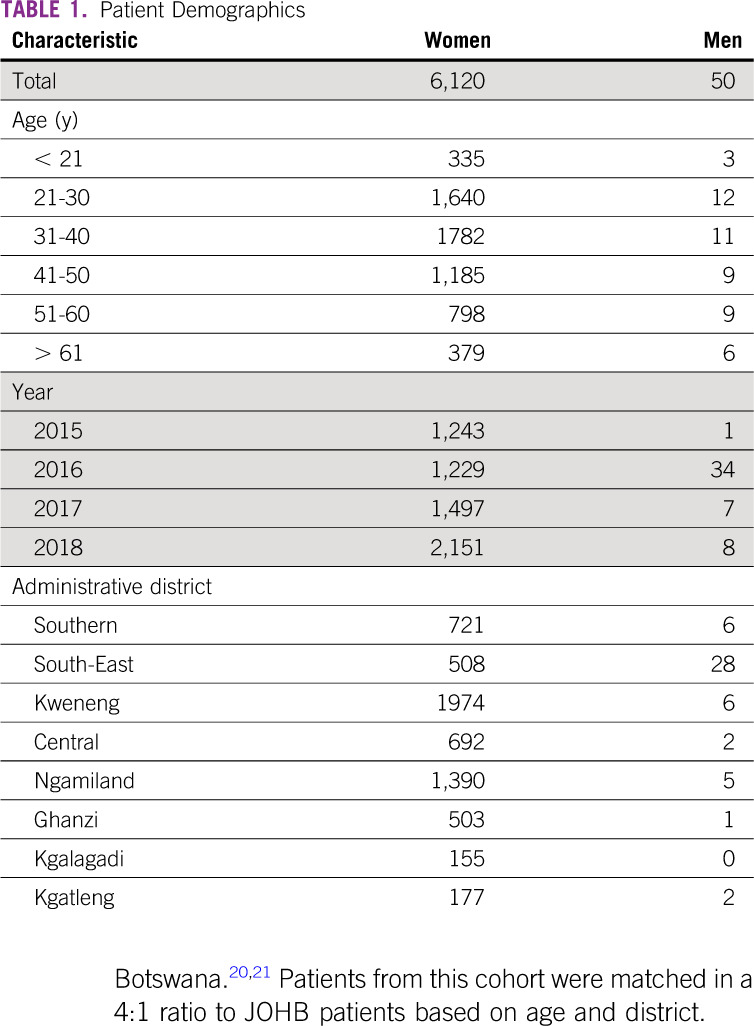
Patient Demographics

### Screening Results

**FIG 2:**
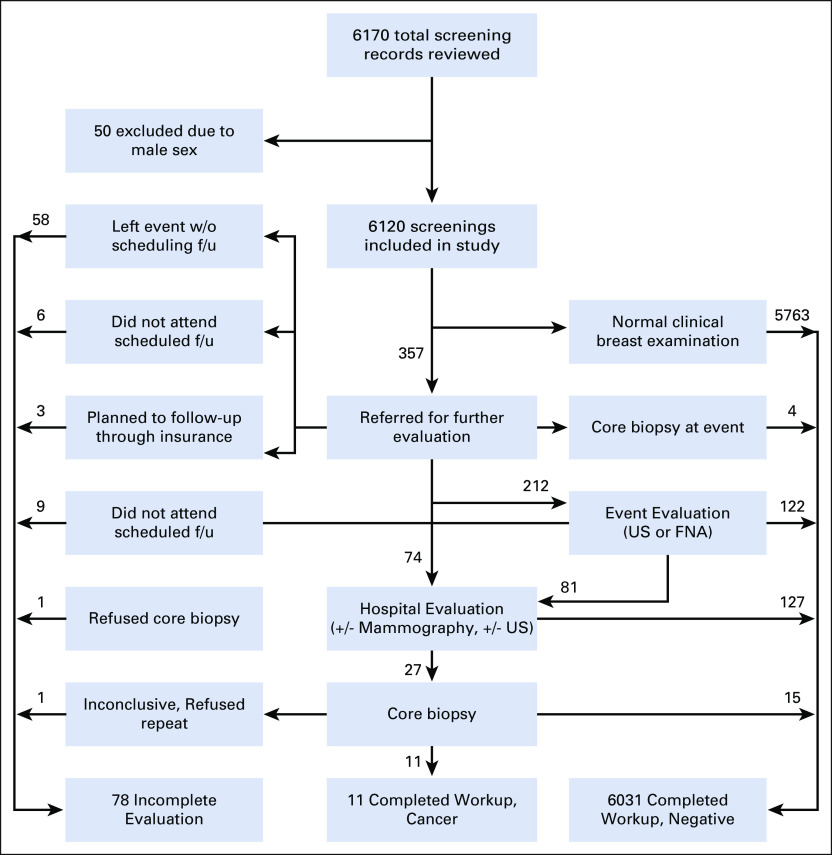
Pathway from screening to diagnosis or loss to follow-up. The results for all women screened were 6,031 negative workups, 11 biopsy-proven cancers, and 78 incomplete evaluations. All arrows going left represent incomplete evaluations, arrows going right represent a completed negative diagnostic evaluation, and arrows down represent further evaluation and eventually a diagnosis of cancer. Women were sent straight to hospital evaluation if they were screened before advanced event evaluation was implemented or if an FNA could not be obtained on-site before ultrasound was available.

Of the 6,120 women screened, 6,042 (98.5%) completed diagnostic evaluation and 78 (1.5%) screened positive on CBE but were lost to follow-up before final diagnosis. The majority of incomplete evaluations occurred because women did not enter the separate queue to see the physician and schedule follow-up at the event (n = 58). Twenty-three (40%) of these women left an event in one large town with long lines and a stockout of t-shirts. There was a nonsignificant trend toward younger women not scheduling evaluation at the event (*P* = .065). The remaining women with incomplete evaluations did not attend scheduled hospital follow-up (n = 17) or followed up through their own insurance and were not tracked by the NGO (n = 3); see Figure 2 for details.

An abnormality requiring further evaluation was present in 357 women, with a 95% CI for referral of 5.83% (5.25%-6.42%) (Table 2 and Fig 2). At presentation, 173 referred women (48.5%) endorsed a breast-related symptom. Women younger than 40 years were more likely to have an abnormality requiring referral than older women, *P* < .01 (Table 2).

**TABLE 2:**
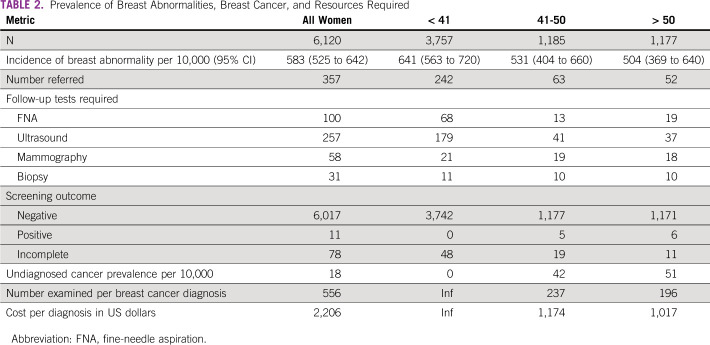
Prevalence of Breast Abnormalities, Breast Cancer, and Resources Required

Of 357 referred women, 122 (34.2%) were able to complete evaluation without hospital referral (98 with ultrasound, 20 through FNA, and 4 through core needle biopsy). Cancer was excluded after hospital evaluation in 142 (39.8%) women (127 with ultrasounds and/or mammogram and 15 with core biopsy).

Eleven women were diagnosed with cancer, six women older than 50 years and five women between 41 and 50 years. The number of women screened to diagnose one breast cancer was 237 (95% CI, 126 to 1910) for women of age 41-50 years and 196 (95% CI, 109 to 977) for women of age older than 50 years. Ten of the 11 reported a breast lump at presentation. Undiagnosed cancer prevalence among all women was 0.18%, 0.42% for women of age 41-50 years, and 0.51% in women of age 51 years and older. No cancers were diagnosed in women younger than 40 years. Women diagnosed with cancer were older than women diagnosed with a benign condition (*P* < .001) or those with incomplete evaluation (*P* < .001).

### Time to Diagnosis

**FIG 3:**
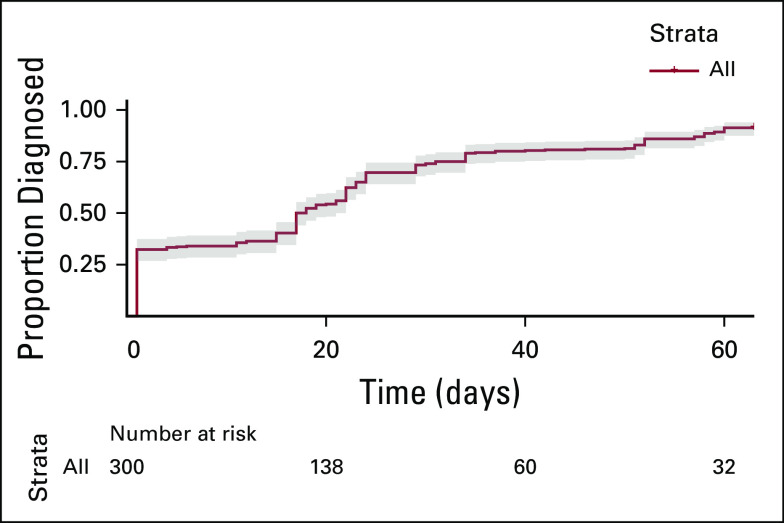
Time to final diagnosis for women who screened positive on clinical breast examination (95% CI).

Median time to diagnosis was 17.5 (1-32.5) days for all women scheduled for follow-up and 34 (19-44) days for women with biopsy-proven cancer (Fig 3). There were no significant differences in time to diagnosis between age groups. A total of 257 ultrasounds, 100 FNAs, 58 mammograms, and 31 biopsies were performed on the referred women (Table 2).

### Cost of Follow-Up Testing

There were no per-unit costs for ultrasounds done at the event. The unit price of hospital ultrasound alone was $64 in US dollars (USD), mammogram plus ultrasound package was $98 USD, FNA reading was $33 USD, and biopsy procedure and analysis was $285 USD. The total cost of hospital follow-up for patients with breast abnormalities was $24,261 USD. This is a follow-up cost of $2,206 USD per breast cancer diagnosed, notably excluding the costs of hosting the event and patient navigation. The follow-up cost per diagnosis for women of age 41-50 years was $1,174 USD and for women of age 51 years and older was $1,017 USD (Table 2).

### Cancer Stage and Outcomes

Ten of 11 women diagnosed with cancer reported symptoms at presentation. The number of women diagnosed with overall stage 0, I, II, and IV disease were 1, 3, 5, and 2, respectively. Tumor stage was not significantly different than from the matched cohort (p = .54). All women received treatment, although only eight initiated treatment immediately following diagnosis and completed the full course. Most women had a favorable prognosis following their treatment course. See Table 3 for more detailed patient information.

**TABLE 3:**
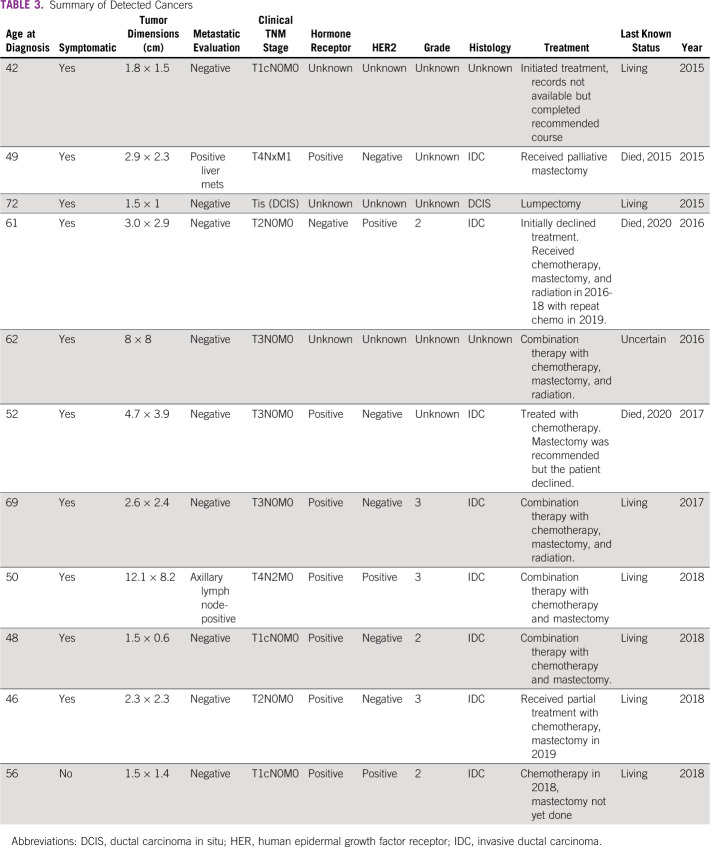
Summary of Detected Cancers

## DISCUSSION

An NGO-led breast cancer screening initiative identified 11 breast cancers among a total of 6,120 previously unscreened women in rural or periurban Botswana. Approximately 5% of women had breast abnormalities detected that required ultrasound, FNA, mammogram, or biopsy for diagnosis. A quarter of women with palpable abnormalities did not complete evaluation, with highest loss occurring with women not scheduling follow-up with a physician at the screening event. It is important to note that nearly half of the referred women and 10 of the 11 women who were diagnosed with cancer reported symptoms at presentation; therefore, the program largely assisted with early diagnosis of symptomatic women and had low yield among asymptomatic women. Additionally, prior studies have shown education as a barrier to seek care and that women who received CBE in the past presented with less delay and lower-stage cancer than the unscreened women so there may be additional long-term benefits that were not captured in this study.^10,24^

Relative to other studies investigating CBEs for breast cancer screening,^11,13,14^ we had a higher proportion of women who screened positive. Given that our study enrolled younger women, and that younger women were more likely to be referred, this difference may be attributable to a higher proportion of benign breast problems such as fibroadenomas in younger women. Our results for cancer prevalence were higher than that found in rural India,^11^ but similar to that described in Sudan^14^ and China^15^ when comparing similar age groups. Our rate is decreased by a large number of women younger than the enrollment criteria for some studies, which was restricted to women older than the age of 35^11^^,^^13^ or 30 years.^12^ The inclusion of symptomatic women in our analysis may contribute to our higher proportion of breast abnormalities and breast cancers than comparable studies, although cluster randomized trials likely included symptomatic women as well. Women with breast symptoms may have been more likely to attend screening events than those without, which could lead to a higher estimate of prevalence. Women were not asked whether symptoms contributed to their motivation to attend. Communities visited by the NGO were more convenient than those where events have not been held, and those unvisited communities may have differing prevalence of breast abnormalities and breast cancer because of limited access to care or differences in lifestyle.

Of note, 82% of incomplete follow-up in our study was because of not scheduling an appointment at a screening event. More than 93% of those who screened positive and scheduled an appointment reached a final diagnosis, which is higher than comparable studies.^11,13-15^ Since socioeconomic factors have been demonstrated to limit follow-up and linkage to care for individuals referred from community-based screening programs,^10,14,25,26^ this high rate of follow-up is likely because of JOHB support for referred individuals, including persistent phone calls and funding all testing, transportation, and accommodations. Our estimates likely overstate the proportion of incomplete evaluations since it includes women who planned to receive evaluation in the private sector, and other women who left events prior to scheduling an appointment may have taken their referral slip to a local public clinic themselves. Loss to follow-up at events highlights the importance of ensuring convenient screening services and streamlining processes such that a separate queue is not required to schedule an appointment. Improving these services would likely substantially decrease loss to follow-up.

The average time to diagnosis in this study is substantially shorter than others published in studies conducted in Botswana. Brown et al^27^ reported median time from first clinic visit to diagnosis for all cancers diagnosed between October 2010 and September 2014 to be 160 (59-653) days. This substantial time difference is probably largely attributable to Journey of Hope patient support, advocacy, and utilization of a private hospital with relatively short wait-times relative to public hospitals.

Despite being annual events, we are unable to assess downstaging of cancer because of the geographic variation and low screening coverage. We would expect that for long-term screening programs of women in the community, both the average number of cancers and cancer stage would decrease. This is because the number of cancers that are prevalent from before the screening interval will decrease, in which case only cancers that are incident over a recent time-scale would be detected.^28,29^ Therefore, we anticipate that the detection rate of breast cancer using CBEs found in this study is higher than what would be expected in a longitudinal CBE program.

Our overall follow-up cost per diagnosis was $2,206 USD for diagnostic evaluation after CBE, although it was lower for older age groups. While this does not include cost of CBE screening itself or patient navigation, testing costs within the public system would be less, and if community nurses or primary care doctors were trained to perform CBE, the unit cost per CBE would also be low. Unfortunately, we cannot make direct comparisons with studies that tested cost per disability-adjusted life years saved.^30^

The stages at diagnosis were comparable for those diagnosed by JOHB compared with the country as a whole. We would expect that given the shorter delay for patient presentation after symptom onset and faster diagnostic evaluation, a larger sample size would likely show lower stage at presentation than the country as a whole. Greater downstaging may be seen in settings with a longer patient-associated delay than Botswana. Larger longitudinal studies in southern Africa may be needed to detect an association between breast cancer screening with CBE and stage at presentation or mortality.

The age of women screened should be based on the efficacy of screening to detect malignancy or premalignancy, with international guidelines targeting women older than 40 or 50 years. Our findings suggest that community-based CBE screening is most efficient for detecting cancers in women older than the age of 40 years. Further study with a larger sample size is required to definitively determine the optimal age range for breast cancer screening, particularly for LMICs with a higher proportion of early-onset, aggressive breast cancers.^31-33^

In conclusion, this study adds to the limited evidence in the region regarding community-based CBE screening, using data from more than 6,000 women. Our findings, which are relevant to similar settings, characterize cancer prevalence in the screened population, number screened per cancer diagnosis, and resource needs for complete diagnostic evaluation. Our results suggest that CBEs may be a reasonable approach to detect breast cancer that is most effective for older and symptomatic women.
